# H/D-Isotope sensitive dual fluorescence of the corrin-ligand of vitamin B_12_†

**DOI:** 10.1039/d4cc06373b

**Published:** 2025-02-04

**Authors:** Steffen Jockusch, Bernhard Kräutler

**Affiliations:** a Department of Chemistry and Center for Photochemical Sciences, Bowling Green State University Bowling Green OH 43403 USA jockus@bgsu.edu; b Institute of Organic Chemistry, University of Innsbruck A-6020 Innsbruck Austria Bernhard.kraeutler@uibk.ac.at

## Abstract

The photoexcited state of the corrin-ligand of vitamin B_12_ is an old puzzle. We show here that the metal-free corrin-ligand emits dual fluorescence in its singlet excited state. As a specific consequence of the asymmetry of the natural corrin-ligand, its strongly emitting singlet excited state exists as a pair of isomers that interconvert rapidly in an unprecedented H/D-Isotope sensitive way in competition with their fluorescent decay.

## Abstract 2

Solving the old puzzle of photoexcited metal-free corrins – dual fluorescence from isomeric singlet excited states undergoing H/D-Isotope sensitive interconversion.

## Graphical abstract

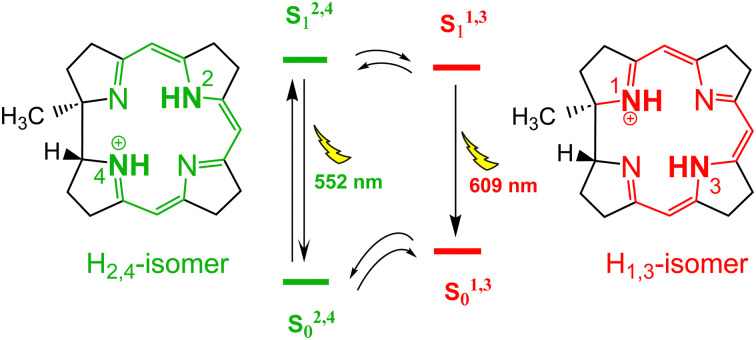

The natural vitamin B_12_ derivatives are intricate cobalt corrins^1,2^ that absorb visible light strongly, but hardly luminesce, as a consequence of de-excitation on the ps timescale.^3^ A first-found natural metal-free corrin^4^ exhibited puzzling steady state luminescence, described as incompatible with a single molecular species.^5^ The scarcity of cobalt-free corrins^6–8^ and the ready isomerization reactions of metal-free B_12_-derivatives^8,9^ have impaired significant progress in gaining insights into the photophysics of such luminescent corrins.^10^ We have resolved this photophysical conundrum in an investigation with the metal-free B_12_-ligand hydrogenobyric acid (Hby),^11^ and by the here delineated discovery of its dual fluorescence, a rare and topical excited state property.^12–14^

Fortunately, natural metal-free corrins have become available recently thanks to modern biosynthetic and biotechnological methodologies, opening up preparative avenues to B_12_-biosynthesis intermediates,^15^ among them hydrogenobyrinic acid *a*,*c*-diamide,^16^ and hydrogenobyric acid (Hby), the metal-free corrin-ligand of vitamin B_12_.^11^ Crystallographic, spectroscopic and computational studies of Hby indicate a single most stable arrangement with a pseudo-diagonal arrangement of two ‘inner’ H-atoms, closely positioned at N2 and N4, de-symmetrizing the approximately *C*_2_-symmetric architecture of the chiral Hby (Scheme 1).^11^ We have studied the luminescence properties of Hby, dissolved in ethanol (EtOH) or perdeuteroethanol (EtOD), by time resolved fluorescence spectroscopy, discovering the dual corrin fluorescence and its H/D-Isotope sensitive cause.

**Scheme 1:**
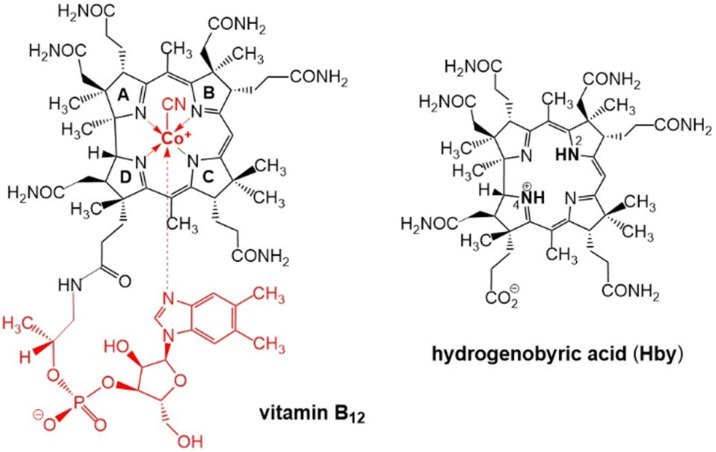
Structural formulas of vitamin B_12_ (left) and of hydrogenobyric acid (Hby), depicted as the 2,4-isomer H_2,4_-Hby.

Absorption and fluorescence spectra of Hby in ethanol are consistent with the existence of a strongly emissive π–π* excited singlet state^11^ exhibiting a characteristically small Stokes shift (of <400 cm^−1^ at 77 K). To investigate the excited state properties of Hby more deeply in EtOH and EtOD at room temperature (296 K) and in frozen solvent matrix at 77 K, steady-state fluorescence spectra were recorded. At room temperature, solutions of Hby in EtOH and EtOD show nearly identical fluorescence emission spectra with peaks at 552 nm and 609 nm, as well as highly similar fluorescence excitation spectra (Fig. 1), which match the absorption spectra (Fig. S1, ESI†). These findings indicate a common ground state origin of the observed emissive states of Hby, and an insignificant equilibrium effect of the solvent-induced H/D-isotopic substitution at the inner corrin nitrogen atoms of Hby. However, at 77 K a strong H/D-Isotope effect was observed in the fluorescence emission spectra of Hby (Fig. 1d), but not on the fluorescence excitation spectra (Fig. 1c). In EtOH matrix, the fluorescence spectrum exhibited peaks at 536 nm and 593 nm (roughly 1 : 3 intensity ratio), as well as a shoulder at 643 nm, resembling the fluorescence at room temperature. Strikingly, in EtOD matrix the fluorescence spectrum of Hby is drastically different from the one in EtOH, and the intensities of the two main emission bands at 536 nm and at roughly 584 nm are reversed to about 2 : 1 (Fig. 1d).

**Fig. 1:**
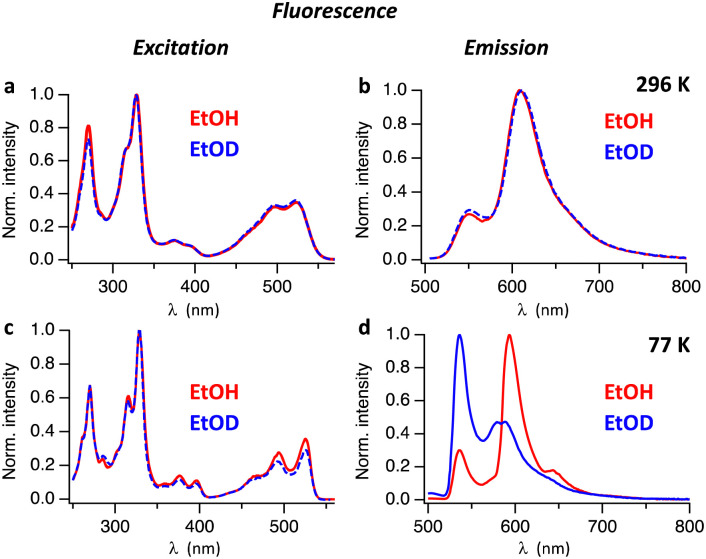
Fluorescence excitation (left) and emission spectra (right) of Hby in EtOH (red) and in EtOD (blue). Top: Spectra recorded at 296 K, (a) fluorescence detection at *λ*_em_ = 606 nm and (b) excitation at *λ*_ex_ = 498 nm. Bottom: Spectra recorded at 77 K, (c) detection of fluorescence at *λ*_em_ = 595 nm and (d) fluorescence from excitation at *λ*_ex_ = 494 nm.

To investigate the origin of the H/D-Isotope effect, we performed time resolved fluorescence measurements, using time-correlated single photon counting. Fig. S2 and S3 (ESI†) show fluorescence decay traces measured with pulsed excitation at 496 nm. At 296 K Hby has a fluorescence lifetime of 3.1 ns in EtOH and 4.6 ns in EtOD. In frozen matrix at 77 K the fluorescence lifetimes increased to 7.5 ns in EtOH and 8.6 ns in EtOD. The time-resolved fluorescence spectra of Hby in EtOH at 77 K reveal a gradual change with time of the ratio between the two main peaks at 536 nm and 593 nm (Fig. 2a–c). At early time scales (red spectrum in Fig. 2b) the peak at 536 nm dominates. At longer delay times, the peak at 593 nm is strongest (blue and green spectra in Fig. 2b). The peak at 593 nm was tentatively assigned to a tautomeric form with a hypothetical structure, discussed below. The rate constant of formation of this tautomer can be estimated from the fluorescence decay trace at 536 nm where the fast component of the bi-exponential decay at 77 K reflects the rate of tautomer formation, *k* ∼ 2 × 10^9^ s^−1^ (Fig. 2c). The time-resolved fluorescence spectra of Hby in EtOD at 77 K show only small changes in the peak ratios of close to 2 : 1, over time, consistent with only a minor contribution by the tautomerization that is now slowed down by a kinetic H/D-Isotope effect of over 10 (Fig. 2d and e).

**Fig. 2:**
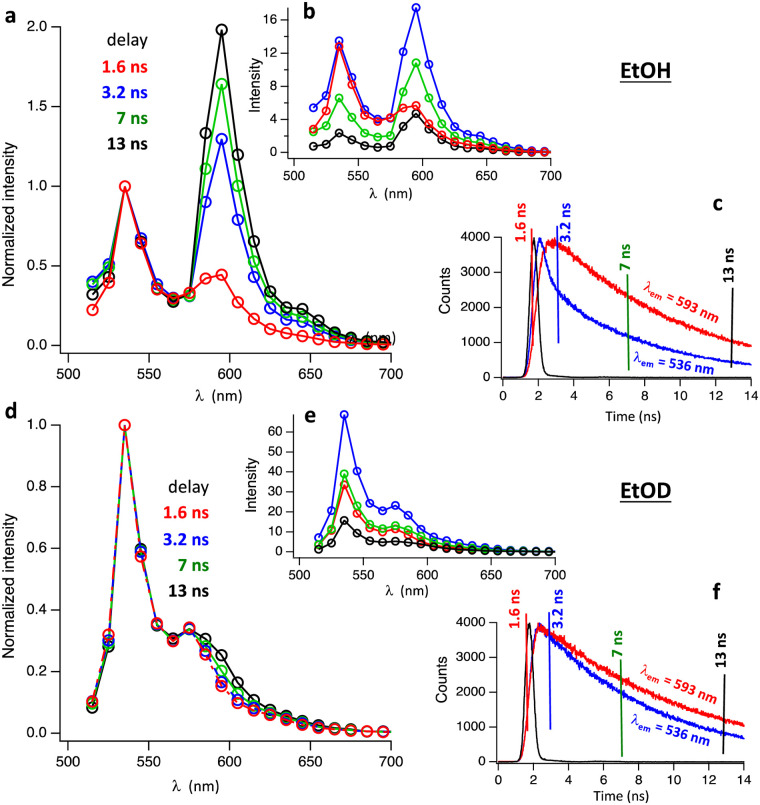
The fluorescence of Hby is strikingly H/D-Isotope sensitive. Time-resolved fluorescence spectra in EtOH (top) and EtOD (bottom) at 77 K (*λ*_ex_ = 456 nm) in absolute intensity (b) and (e) and normalized at 536 nm (a) and (d). The delay times are marked in the fluorescence decay traces (c) and (f) monitored at *λ*_em_ = 536 nm (blue) and *λ*_em_ = 593 nm (red). Note that time zero is set to ∼1 ns before the start of the excitation pulse. The instruments response function is shown in black (c) and (f).

The fluorescence spectra of Hby in EtOH show an apparent two-band structure at 296 K and 77 K with an approximate 1 : 3 intensity ratio,^11^ and maxima separated by 1696 cm^−1^ (296 K) or 1794 cm^−1^ (77 K), *i.e.* by significantly larger energy differences than typical of a corrin vibrational progression.^3,10,17^ At 296 K, solutions of Hby in EtOH as well as in EtOD show weaker fluorescence emission at 552 nm from the directly excited singlet state S_1_^2,4^ that retains the ground state bonding of X_2/4_-Hby (with X = H or D at N2 and N4). The more intense emission at 609 nm in EtOH at 77 K indicates a second excited singlet state (S_1_^1,3^) corresponding to the hypothetical H_1/3_-tautomer of Hby (see Scheme 2). It features a deduced vibrational spacing of close to 1300 cm^−1^, as also shown by the now more intense F_2,4_ of Hby in EtOD (*i.e.* of Hby(DD)), from complete H/D-exchange. In EtOH : EtOD = 1 : 1 the two fluorescence maxima displayed relative intensities of about 1 : 1.2 at 77 K (Fig. S4, ESI†), reflecting partial deuteration of Hby.

**Scheme 2:**
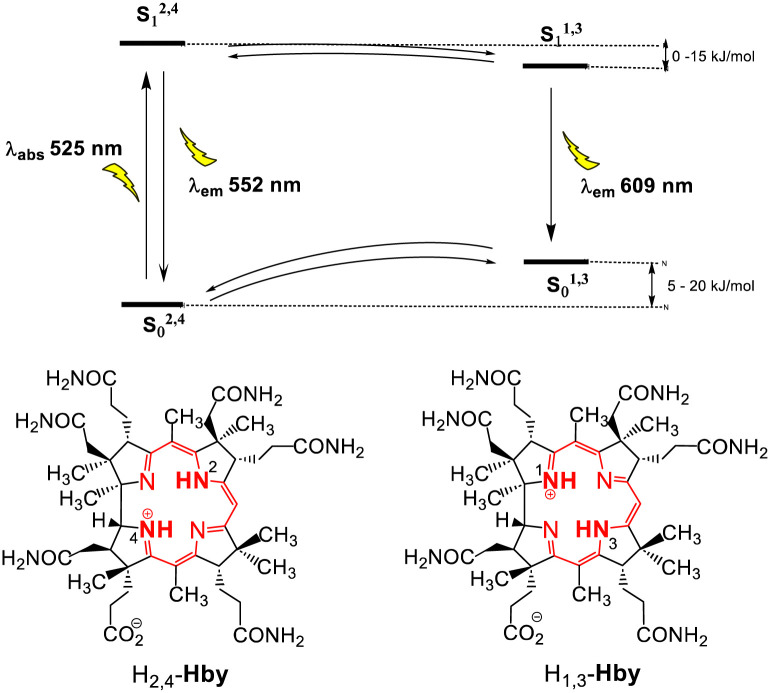
Energy diagram with tautomers H_2/4_-Hby and H_1/3_-Hby in the ground states S_0_^2,4^ and S_0_^1,3^, as well as the respective excited states, S_1_^2,4^ and S_1_^1,3^, undergoing interconversion by thermal activation. Estimated relative ground state and relative singlet excited state energies, and experimental absorption and emission wavelengths are also shown.

We assign the observed intensity reversal of F_2,4_ and F_1,3_ at 77 K to a large kinetic H/D-Isotope effect on the rate of conversion of the directly excited state S_1_^2,4^ of Hby to the slightly more stable, isomeric excited singlet state S_1_^1,3^. This hypothetical tautomer is provisionally assigned here to the pseudo-diagonal alternative X_1/3_-Hby, with X = H or D at the inner nitrogen atoms N1–N3. Our structural studies^11^ have not excluded very minor contributions of alternative tautomeric ground state structures, *e.g.*, of H_1/3_-Hby, with ‘inner’ protons at N1 and N3. Indeed, the crystal structure of the metal-free HCor^18^ has provided formal precedence for the N1–N3 tautomer (H_1/3_-HCor) of this synthetic model corrin.^6^ The specific non-linear *trans*-annular H-bonding from the H-donors N2 and N4 to the most closely placed H-bond accepting N-atoms N1 and N3, respectively, is revealed by the crystallographic data for Hby.^11^ These intramolecular neighborhood relations may provide a most probable path for a rapid ground state equilibration of the more stable N2–N4-protomer H_2/4_-Hby with the corresponding hypothetical N1–N3 tautomer H_1/3_-Hby. NMR-data of Hby indicated rates of up to only 10^2^–10^3^ s^−1^ for thermal exchange reactions of HN4 and HN2 at around 300 K,^11^ and insignificant H/D-isotopic fractionation at these N-atoms in the aqueous solution.^19^

The two tautomeric singlet excited states of Hby interconvert remarkably fast in EtOH, outcompeting effectively their fluorescence decay, with common fluorescence lifetimes estimated as 3.1 ns at 296 K (Fig. S3, ESI†), and 7.5 ns at 77 K. At 77 K in EtOH, the forward isomerization still occurs with an estimated rate of about 2 × 10^9^ s^−1^. At 296 K in EtOD the two singlet excited states of Hby also rapidly equilibrate and feature a common effective lifetime of 4.6 ns (Fig. S3, ESI†). At 77 K, the excited singlet state S_1_^2,4^ of D_2/4_-Hby in EtOD emits fluorescence at around 593 nm with a fluorescence lifetime of 8.6 ns. However, in contrast to the situation of Hby in EtOH, at this low temperature S_1_^2,4^ of D_2/4_-Hby interconverts at least 10 times slower than H_2/4_-Hby, with its second excited state S_1_^1,3^ (of D_1/3_-Hby), emitting near 600 nm. In conclusion, the observations of the fluorescence of Hby at 296 K and at 77 K, in EtOH as well as in EtOD, furnish a diagram with two tautomeric states in both, the singlet excited and ground state manifolds of Hby (see Scheme 2). The two tautomeric forms equilibrate in a kinetically H/D-Isotope sensitive process, proposed here to occur *via* an intramolecular movement of two hydrogen atoms (H or D). Thus, the derived existence of the two strongly emissive tautomeric singlet excited states S_1_^2,4^ and S_1_^1,3^ explains the observed phenomenon of dual fluorescence. Interestingly, the predominant tautomeric structure H_2,4_-Hby of Hby is similarly displayed by the natural metal-free corrins hydrogenobyrinic acid *a*,*c*-diamide^16^ and hydrogenobalamin,^20^ both also featuring the here further scrutinized exceptional 2-band pattern of their fluorescence emission spectra.

The ground state S_0_^2,4^ of the metal-free corrin Hby is calculated more stable by 4 kJ mol^−1^ (gas phase) or about 20 kJ mol^−1^ (in implicit polar solvent) than the lowest energy alternative tautomer, H_1/3_.^11^ As derived from the energies of the two observed main emissions at 552 nm and at 609 nm, the two corresponding excited singlet states S_0_^2,4^ and S_0_^1,3^ of Hby are situated about 220 or 200 kJ above their respective isomeric ground states S_0_^2,4^ and S_0_^1,3^. The calculated stability difference of S_0_^2,4^*vs.* S_0_^1,3^ of about 20 kJ mol^−1^ (implicit solvent), roughly matches the energy difference of the fluorescence emissions F_2,4_ and F_1,3_, thus, positioning the two isomeric singlet excited states S_1_^2,4^ and S_1_^1,3^ at only barely differing energy levels. Still, the excited H_1/3_-state S_1_^1,3^ tends to be calculated at a slightly deeper level than the isomer S_1_^2,4^ from direct light excitation of Hby (see Scheme 2). This estimation of the key energy levels correlates qualitatively with the intensities of the fluorescence emissions under the conditions of extensive equilibration of the two singlet excited states. They show a 1 : 3 ratio in favor of the emission F_1,3_ from the only indirectly populated singlet excited state S_1_^1,3^, here associated with a H_1/3_-structure.

The tentative structural assignment of the H_1/3_-type to the excited singlet state S_1_^1,3^ of Hby invokes a singlet excited state isomerization with migration of two hydrogen atoms (H or D, depending upon the solvent deuterium content). For porphyrins, in which a diagonal arrangement of the ‘inner’ H-atoms predominates by far, a stepwise two H-atom isomerization has been established, featuring large kinetic H/D-Isotope effects.^21^ In less symmetric cyclic tetrapyrroles, such as porphycenes,^22^ free-base corroles^14^ and an oxaporphyrinium cation,^13^ tautomeric singlet excited states exist, which rapidly interconvert *via* a single or double H-atom migration, and show dual emission. On the other hand, the H_2/4_- and H_1/3_-isomers of the skewed, helical metal-free corrin Hby represent two non-identical ground state tautomers with pseudo-diagonal position of the ‘inner’ N-bonded H-atoms and with a seemingly cryptic difference of their chemical constitution. The H_2/4_-form and the less stable hypothetical H_1/3_-isomers of Hby are calculated to only differ by <20 kJ mol^−1^ in their relative stability.^11^ Computer-based theoretical deductions concerning the changes in the corrin molecule upon photoexcitation may help to identify the driving force for stabilizing the hypothetical H_1/3_-structure in the excited singlet state S_1_^1,3^ over its H_2/4_-tautomer S_1_^2,4^. Clearly, the π-type interactions of the corrin system in the lowest excited singlet state and in the ground state are significantly different, allowing for the altered stability order of H_2/4_*vs.* H_1/3_. The here deduced fast equilibration of two lowest excited singlet states *via* tautomerization is subject to a primary kinetic H/D-Isotope effect of >10 and probably occurs *via* a step-wise migration of two hydrogen atoms. In fact, the here presented study also helps to rationalize the puzzling observations^5,23^ of seemingly aberrant fluorescence spectra of Toohey's^4^ metal-free corrin, and then ascribed to the presence of a probable impurity.^5^

In summary, this first dedicated study of the fluorescence properties of a well-characterized metal-free corrin has given unprecedented insights into the properties of the low lying π–π* excited singlet states of the natural corrin ligand, unperturbed by a coordinated metal. It revealed, first-of-all, a striking dual fluorescence of the asymmetric corrinoid B_12_-ligand Hby. This observation for the excited singlet state of the metal-free corrin Hby provides evidence for the fleeting existence of two strongly emissive states that feature a common type of corrin π-system. As proposed here, they interconvert by a very fast adiabatic excited state tautomerization, subject to a striking kinetic H/D-Isotope effect.

Clearly, the properties of the excited states of corrins has remained a remarkably challenging subject.^3–5,24,25^ The surprising discovery of a photoregulatory role of coenzyme B_12_,^26^ widely important in bacteria,^27^ has provided the photochemistry of vitamin B_12_ derivatives with a remarkable biological relevance.^3,25,28,29^ Meanwhile, the so gained insights were applied to the fields of optogenetics and synthetic biology.^29–31^ Along such lines, the fluorescence of natural metal-free corrins, their here described dual fluorescence,^12^ as well as its early detected fluorescence polarization,^23^ may be specific new B_12_-related assets in the growing field of well-studied and useful fluorescent proteins.^32–35^ The intricate and structurally complex natural B_12_ derivatives clearly continue to fascinate,^1,2,36^ and to provide new opportunities in fundamental and applied chemical and biological research.^37–39^

We are grateful to Evelyne Deery and Martin J. Warren for samples of hydrogenobyric acid. We also thank the Austrian Science Fund FWF (project P 33059 to B. K.) for financial support of this work.

## Data availability

The data supporting this article have been included as part of the ESI.†

## Conflicts of interest

There are no conflicts to declare.

## Supplementary Material

CC-061-D4CC06373B-s001
