# Supplementary material for: H/D-Isotope sensitive dual fluorescence of the corrin-ligand of vitamin B12
Source: Chem Commun (Camb). 2025 Feb 4;61(19):3904–7. doi: 10.1039/d4cc06373b (PMC11816046; doi:10.1039/d4cc06373b)
Supplement: CC-061-D4CC06373B-s001 [file CC-061-D4CC06373B-s001.pdf]

Supplementary Information (SI) for ChemComm.
This journal is © The Royal Society of Chemistry 2025

H/D-Isotope Sensitive Dual Fluorescence of the Corrin-Ligand of Vitamin B_{12}

Steffen Jockusch[1]* and Bernhard Kräutler[2]*

[1] Department of Chemistry and Center for Photochemical Sciences, Bowling Green State University, Bowling Green, OH 43403 (USA), E-mail: jockus@bgsu.edu

[2] Institute of Organic Chemistry, University of Innsbruck, 6020 Innsbruck (Austria), E-mail: bernhard.kraeutler@uibk.ac.at

Supplementary Information

Materials and Methods.

Hydrogenobyric Acid (**Hby**) was prepared as described previously.[1] Spectroscopic grade ethanol (EtOH) from Sigma Aldrich and perdeuteroethanol (EtOD) from Cambridge Isotope Laboratories were used, as received.

UV-Vis absorption measurements were performed on an Agilent 8453 spectrometer. Steady-state fluorescence spectra were recorded on a HORIBA Jobin Yvon Fluorolog-3 fluorescence spectrometer. Time-resolved fluorescence measurements were performed on an Edinburgh Analytical Instruments FLS1000 fluorescence spectrometer in conjunction with an EPL 450 pulsed diode laser for excitation at 456 nm, or on an Edinburgh Analytical Instruments OB920 fluorescence spectrometer in conjunction with a PicoQuant pulsed LED for excitation at 496 nm. Room temperature measurements (296 K) were performed in 1x1 cm quarts cuvettes. For measurements in frozen matrix at 77 K, sample solutions were placed in 4 mm (inner diameter) quartz tubes and immersed into liquid nitrogen in an optical quartz dewar which was mounted inside the fluorescence spectrometer.

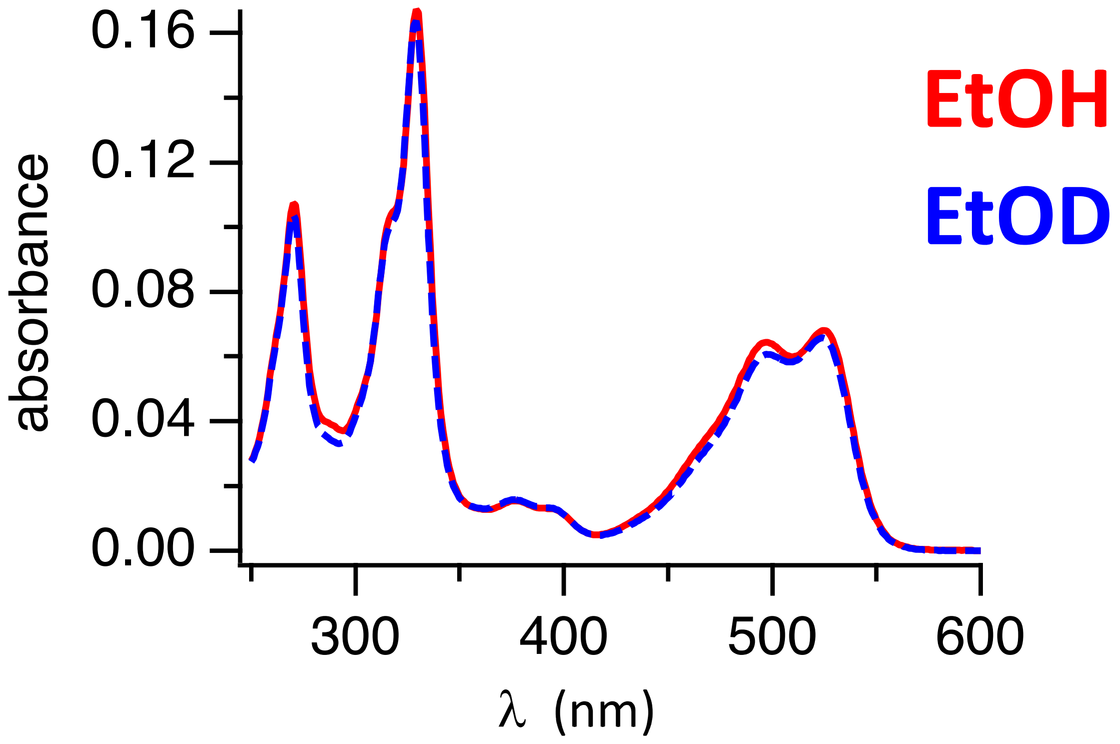

Fig. S1. UV-vis absorption spectra of **Hby** in EtOH (red) and EtOD (blue) at 296 K.

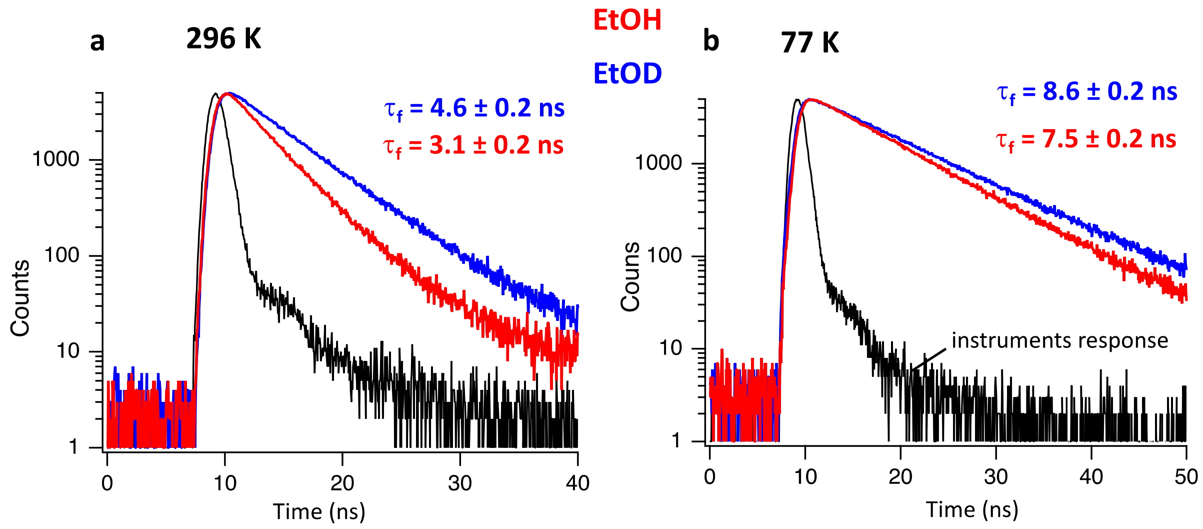

Fig. S2. Fluorescence decay traces of **Hby** in EtOH (red) and EtOD (blue) at 296 K (a) and 77 K (b). λ_{ex} = 496 nm; λ_{em} = 609 nm (a), λ_{em} = 593 nm (b). The instruments response function is shown in black.

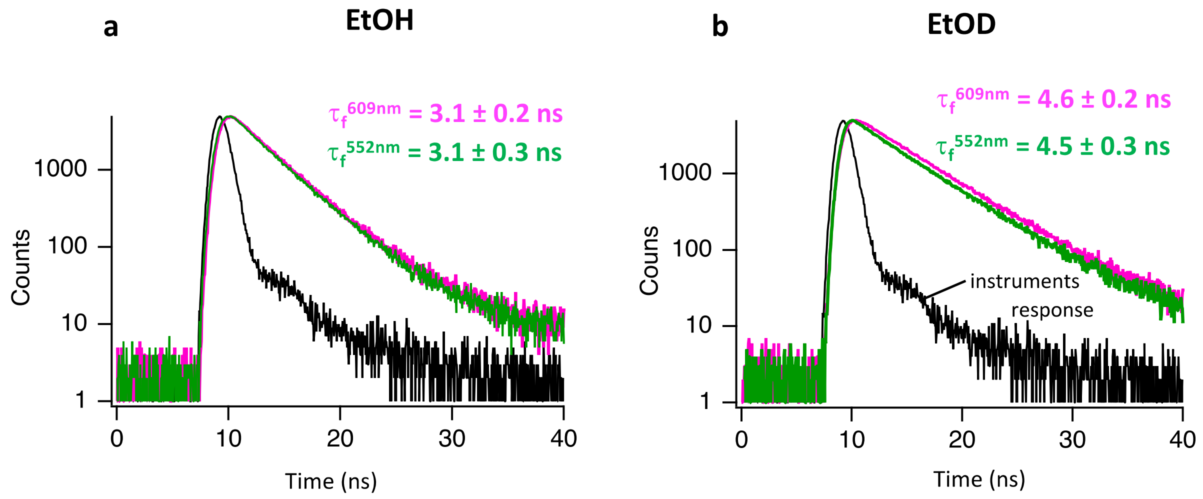

Fig. S3. Fluorescence decay traces of **Hby** in EtOH (a) and EtOD (b) at 296 K. λ_{ex} = 496 nm; λ_{em} = 609 nm (magna), λ_{em} = 552 nm (green). The instruments response function is shown in black.

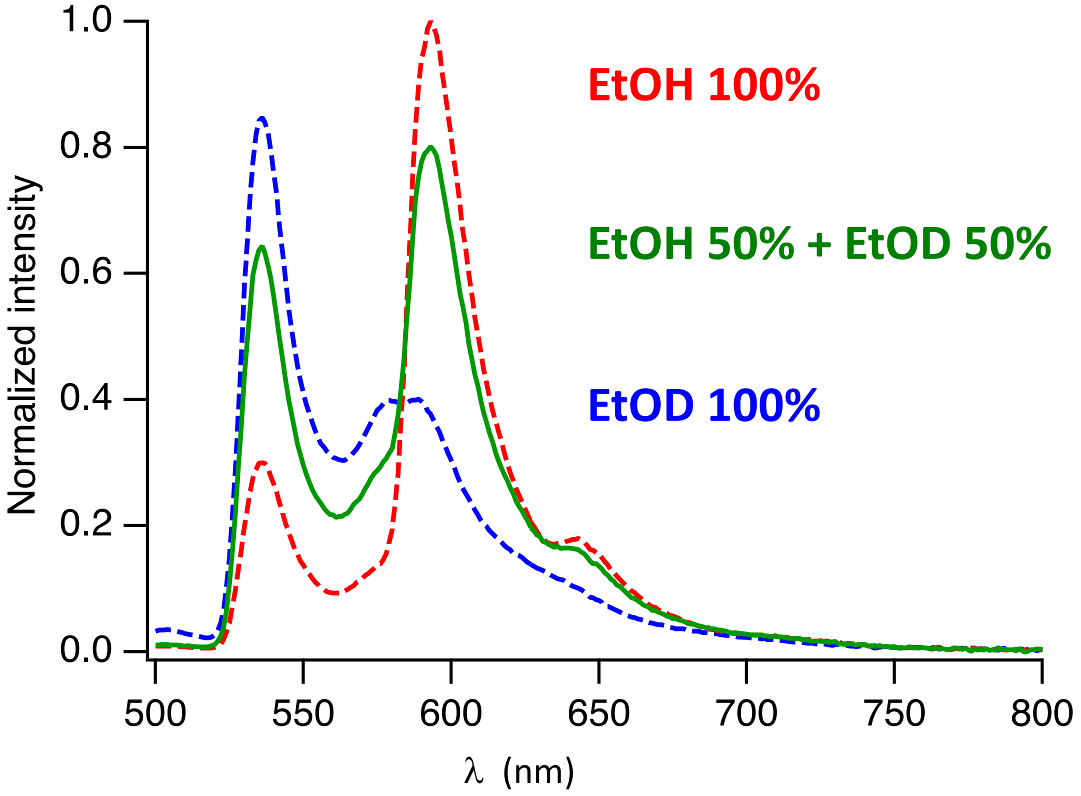

Fig. S4. Fluorescence spectra of **Hby** in EtOH (red), EtOD (blue) and a mixture of 50% EtOH and 50% EtOD (green) at 77 K with excitation at λ_{ex} = 494 nm.

References

[1] C. Kieninger, E. Deery, A. D. Lawrence, M. Podewitz, K. Wurst, E. Nemoto-Smith, F. J. Widner, J. A. Baker, S. Jockusch, C. R. Kreutz, K. R. Liedl, K. Gruber, M. J. Warren, B. Kräutler, *Angew. Chem. Int. Ed.* **2019**, *58*, 10756-10760.
